# A new South American darter (Crenuchidae: *Characidium* ) from rivers draining the Northeastern Mata Atlantica Freshwater Ecoregion, Brazil: morphological and molecular evidence

**DOI:** 10.1111/jfb.70316

**Published:** 2025-12-23

**Authors:** Angela M. Zanata, Leonardo Oliveira‐Silva

**Affiliations:** ^1^ Programa de Pós‐Graduação em Biodiversidade e Evolução Instituto de Biologia, Universidade Federal da Bahia Salvador Brazil; ^2^ Departamento de Biologia Estrutural e Funcional Instituto de Biociências, Universidade Estadual Paulista São Paulo Brazil

**Keywords:** genetic divergence, integrative taxonomy, vestigial swimbladder

## Abstract

*Characidium tupi*, a new species from rivers draining the southern portion of the Northeastern Mata Atlantica Freshwater Ecoregion, is described. The new species can be distinguished from all congeners by its colour pattern, including 7–14 dark bars enlarged and more conspicuous on their ventralmost portion, resulting in a longitudinal series of conspicuous dark blotches positioned around the midlateral line of the body, and the presence of a conspicuous 3‐shaped black band approximately at the midlength of the caudal‐fin rays. In addition, the new species differs from most congeners by having a dark band below the midlength of the dorsal‐fin rays initiating on the third or fourth branched rays and isthmus, and large areas around the pectoral‐fin bases scaleless, but with a broad central patch of scales. Both sexes possess a vestigial swimbladder, which was slightly longer in the males examined. Species delimitation analyses were based on the mitochondrial cytochrome c oxidase subunit I marker and the genetic distances obtained reinforce the recognition of *C. tupi* as a distinct species, with divergences exceeding 6% in relation to its closely related species.

## 1 INTRODUCTION

Eight freshwater ecoregions are recognized by Abell et al. (2008) to comprise river basins draining the eastern Brazilian coast, in addition to the São Francisco ecoregion. The Northeastern Mata Atlantica Freshwater (NMAF) ecoregion stands out as the widest in latitude range and includes at least 25 independent river basins (e.g. Paraguaçu, Contas, Pardo, Doce, Jequitinhonha), draining parts of four Brazilian states (i.e. Bahia, Espírito Santo, Sergipe and Minas Gerais). Although these rivers have been sampled for centuries, their ichthyofauna can still be considered far from being fully known because new species are described practically every year in the last decades (see Fricke et al., 2025). Furthermore, taxa yet to be precisely identified to the species level have been appearing in recent publications dealing with the ichthyofauna of rivers draining the ecoregion (e.g. Silva et al., 2020; Vieira‐Guimarães et al., 2024).

Coastal Brazilian drainages are species‐rich in terms of *Characidium* Reinhardt, with 25 of the 87 valid species originally described to such basins, most of them endemic to one or a few of these drainages (Fricke et al., 2025; Toledo‐Piza et al., 2024). Most of these species were described relatively recently, with five of them described in the last decade from rivers included in the NMAF ecoregion (i.e. *C. clistenesi* Melo & Espíndola, *C. cricarense* Malanski et al., *C. deludens* Zanata & Camelier, *C. helmeri* Zanata, Sarmento‐Soares & Martins‐Pinheiro, *C. kamakan* Zanata & Camelier, *C. krenak* Oliveira‐Silva et al.). Three other congeners were originally described from the ecoregion in the past (i.e., *C. bahiense* Almeida, *C. samurai* Zanata & Camelier, *C. timbuiense* Travassos). In addition, *C. bimaculatum* Fowler, described from Fortaleza, Ceará State, occurs in the ecoregion (Melo & Espíndola, 2016).

The endemic nature of the ichthyofauna of the NMAF ecoregion have been pointed out in a series of studies, with groups of basins or bioregions within the ecoregion defined on the basis of exclusively shared species (Camelier & Zanata, 2014; Vieira‐Guimarães et al., 2024). For instance, *C. bahiense* is one of the species that is exclusive of a group of drainages named the North Group by Camelier and Zanata (2014). Also, according to Vieira‐Guimarães et al. (2024), the presence of *C. cricarense* and *C. timbuiense* is exclusive of and supports the Coastal Tablelands Bioregion and Southern Bioregion, respectively. Some other species of the genus are considered endemic from a particular river basin within the NMAF ecoregion (e.g. *C. clistenesi* from rio Paraguaçu, *C. kamakan* from rio Pardo, *C. krenak* from rio Doce).

A relatively broad integrative study by Oliveira‐Silva et al. (2024), investigating the taxonomic boundaries of species of *Characidium* within the genus in rivers draining the northeastern region of Brazil, not only confirmed the previously known species in the NMAF ecoregion but also identified morphotypes hypothesized as new species for the region, supported by both morphological and molecular data. One of these morphotypes was examined in the present study and its status as a new species within the genus was confirmed. The new species, apparently endemic to the South Group of Camelier and Zanata (2014) and Coastal Tablelands Bioregion of Vieira‐Guimarães et al. (2024), is described herein.

## 2 MATERIALS AND METHODS

Most of the specimens of the new species examined were in existing museum collections at Universidade Federal da Bahia (UFBA), Bahia, Brazil and Museu de Zoologia da Universidade de São Paulo, São Paulo, Brazil. Some of the specimens used in the morphological and molecular analyses were collected under licence number 13754‐7, issued by the Brazilian Institute for the Environment and Renewable Natural Resources. The field studies did not involve endangered species.

### 2.1 Morphological analyses

Counts and measurements were taken according to Buckup (1993a), Melo and Oyakawa (2015), and Zanata et al. (2023). Measurements were taken with digital callipers to the nearest 0.1 mm and expressed as percentages of standard length (*L*_S_), except subunits of the head, which are given as percentages of head length (*L*_H_). In the list of paratypes, asterisks indicate lots for which measurements are included in Table 1. Meristic data are given in the description, an asterisk indicates counts of the holotype and the frequency of each count is given in parentheses. Counts of vertebrae, branchiostegal rays, procurrent caudal‐fin rays, epurals and other osteological observations were made only in cleared and stained (c&s) paratypes, prepared according to the method of Taylor and Van Dyke (1985). Dentary teeth were counted in c&s specimens and in the largest alcohol‐preserved specimens. For analysis of the swimbladder, specimens had their abdominal cavity exposed through a lateral section, on the right side, and the swimbladder was removed. The swimbladder length was measured from the anteriormost to the posteriormost margin of the first chamber, and from the anteriormost margin to the posterior tip of the second chamber. Measurements were given as percentages of standard length (*L*_S_), except when noted. The pattern of *circuli* and *radii* were observed on scales situated between the dorsal‐fin base and lateral line, after being stained in alizarin. The pseudotympanum morphology was examined after the removal of the overlying skin, adipose tissue and lateral‐line nerve of alcohol‐preserved specimens. Institutional abbreviations follow Fricke and Eschmeyer (2025).

### 2.2 Molecular data and analyses

Genomic DNA was extracted from muscle tissue using the Wizard Genomic DNA Purification Kit (Promega) following the manufacturer's protocol. PCR conditions were optimized to amplify a fragment of the mitochondrial cytochrome c oxidase subunit I (COI) gene using the primers FishF1 and FishR1 (Ward et al., 2005). PCR reaction, purification and sequencing were prepared following Oliveira‐Silva et al. (2024). In the present study, six new sequences were generated and incorporated into the alignment defining the F clade of the species *Characidium* proposed by Oliveira‐Silva et al. (2024). These included sequences attributed to the newly identified species from the rio São Mateus, as well as two additional sequences from the morphotype *Characidium* sp. 1, collected in the rio Doce. A sequence of *Characidium gomesi* Travassos from GenBank was used as the outgroup for phylogenetic inference. Details on sequences and GenBank and BOLD accession numbers are provided in Table S1. Sequence assembly was conducted using Geneious 7.1.4 (Kearse et al., 2012) and alignment was performed using MUSCLE (Edgar, 2004) under default parameters. Following alignment, the matrix was manually inspected in MEGA v.11 (Tamura et al., 2021) for corrections where necessary, and sequences were validated through translation into amino acids. Substitution saturation (Iss) was assessed using DAMBE v5.3.38 (Xia, 2013). Genetic distances were estimated across all specimens, as well as at intra‐ and interspecific levels, using 1000 bootstrap replicates in MEGA v.11 (Tamura et al., 2021), with no root assumption. Nucleotide variation and substitution patterns were also analysed in MEGA v.11.

Maximum likelihood (ML) phylogenetic reconstruction was performed using RAxML PTHREADS‐SSE3 v.8 (Stamatakis, 2014) under the GTRGAMMA model, employing five random parsimony trees on the Zungaro server at LBP‐UNESP. The best‐scoring ML tree was obtained from 10 independent searches, with node support evaluated through 1000 bootstrap replicates using the autoMRE function with bootstopping criteria (Pattengale et al., 2010).

Molecular species delimitation was assessed using Assemble Species by Automatic Partitioning (ASAP; Puillandre et al., 2021) and the Bayesian implementation of the Poisson tree process (bPTP; Zhang et al., 2013). ASAP analyses were performed via the web server (https://bioinfo.mnhn.fr/abi/public/asap/asapweb.html) using the Kimura 2‐parameter (K80; 2.0) model. The bPTP analysis utilized the best ML tree as input, underwent 100,000 generations, and was conducted under default settings on the bPTP web server (http://species.h-its.org/ptp/). Overall and pairwise genetic distances were computed under the Kimura 2‐parameter + gamma model (K2P+Γ) in MEGA v.11, with taxon order determined according to ASAP and bPTP results. Outgroup taxa were excluded from ASAP, bPTP and genetic distance analyses.

## 3 RESULTS

### 3.1 *Characidium tupi*, new species

urn:lsid:zoobank.org:pub:AD5B5D4A‐062F‐4132‐AF60‐6CBC20A0DDF0.

urn:lsid:zoobank.org:act:FC360BCD‐E970‐4F0C‐B20F‐B35607E95950.

**FIGURE 1:**
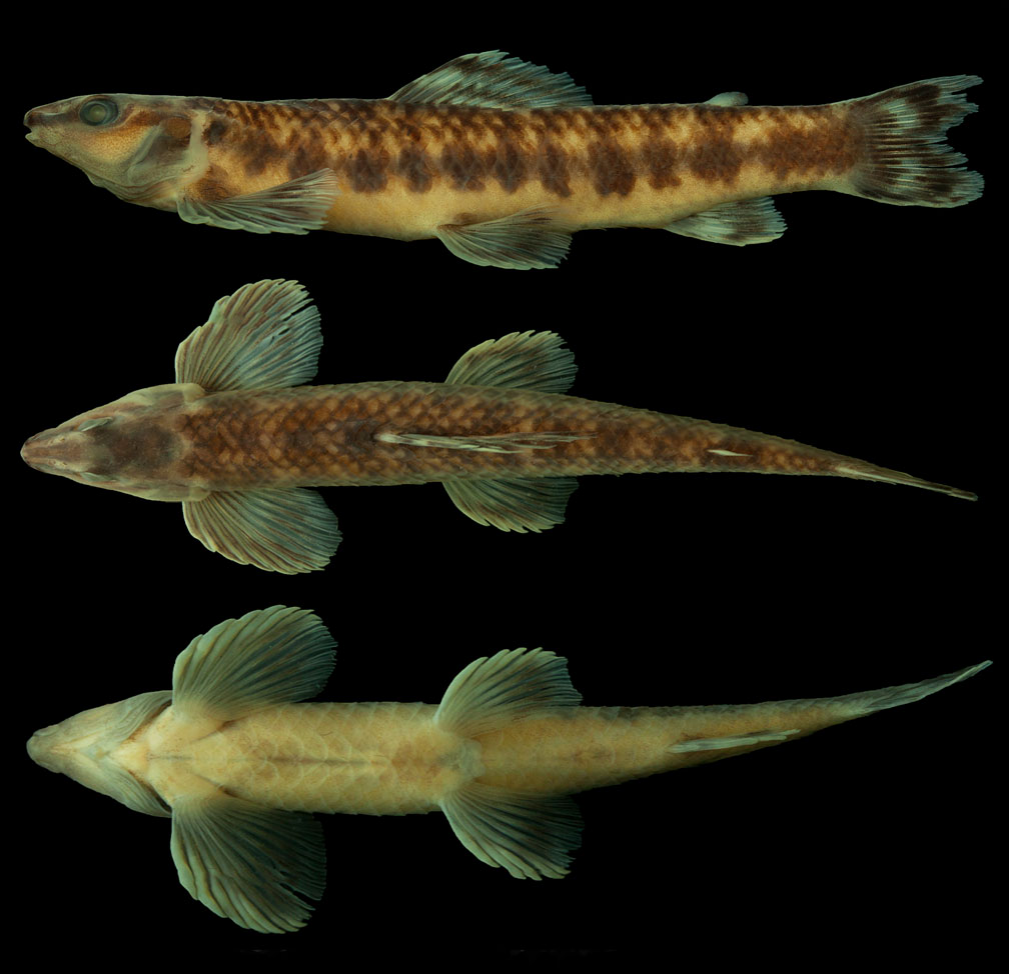
*Characidium tupi*: MZUSP 130914, holotype, 71.7 mm *L*_S_, dorsal, lateral and ventral views, Brazil, Bahia, Teixeira de Freitas, rio Itanhém, near Povoado Prainha.

**FIGURE 2:**
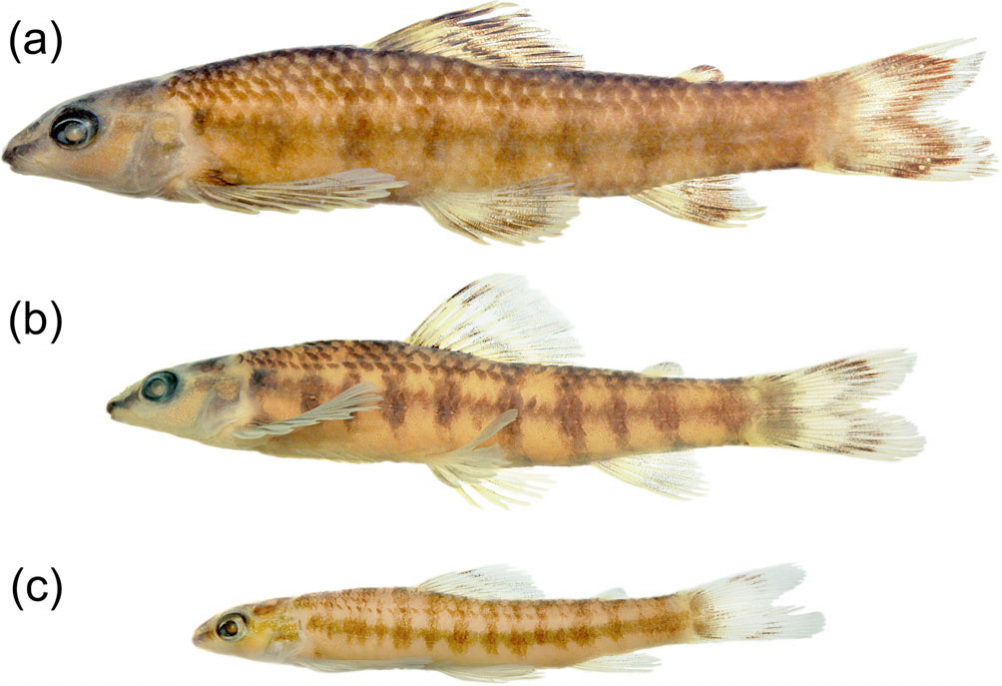
Paratypes of *Characidium tupi*, lateral view. (a) UFBA 4819, 53.3 mm *L*_S_, stream of the rio Braço Sul, rio Jucuruçu basin, Bahia state; (b) UFBA 9946, 46.4 mm *L*_S_, rio Prado, rio Jucuruçu basin, Minas Gerais state. (c) UFBA 4819, 38.4 *L*_S_ mm, same locality as above in (a).

**FIGURE 3:**
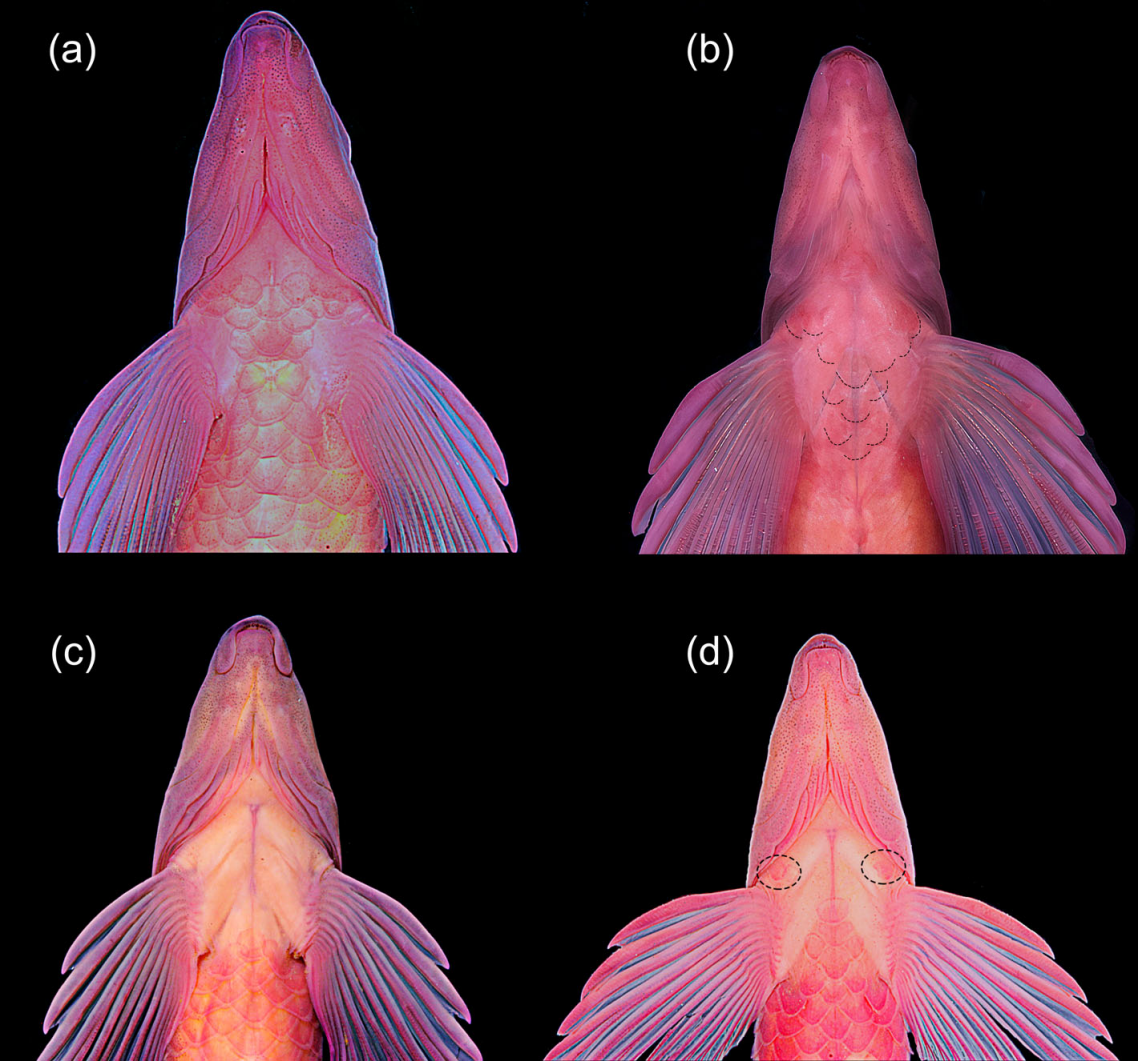
Ventral view of the anterior portion of body, illustrating the distribution of scales on the area of *Characidium tupi*: (a) UFBA 8766, paratype, 58.9 mm *L*_S_, rio dos Frades basin, Bahia state; (b) UFBA 4969, 47.5 mm *L*_S_, rio Jucuruçu basin, Bahia state; and *Characidium alipioi*: (c, d) UFBA 9939, 55.0 and 46.5 mm *L*_S_, respectively, rio Pomba, rio Paraíba do Sul basin, Minas Gerais state; dotted circles indicate isolate scale anteriorly positioned.

**FIGURE 4:**
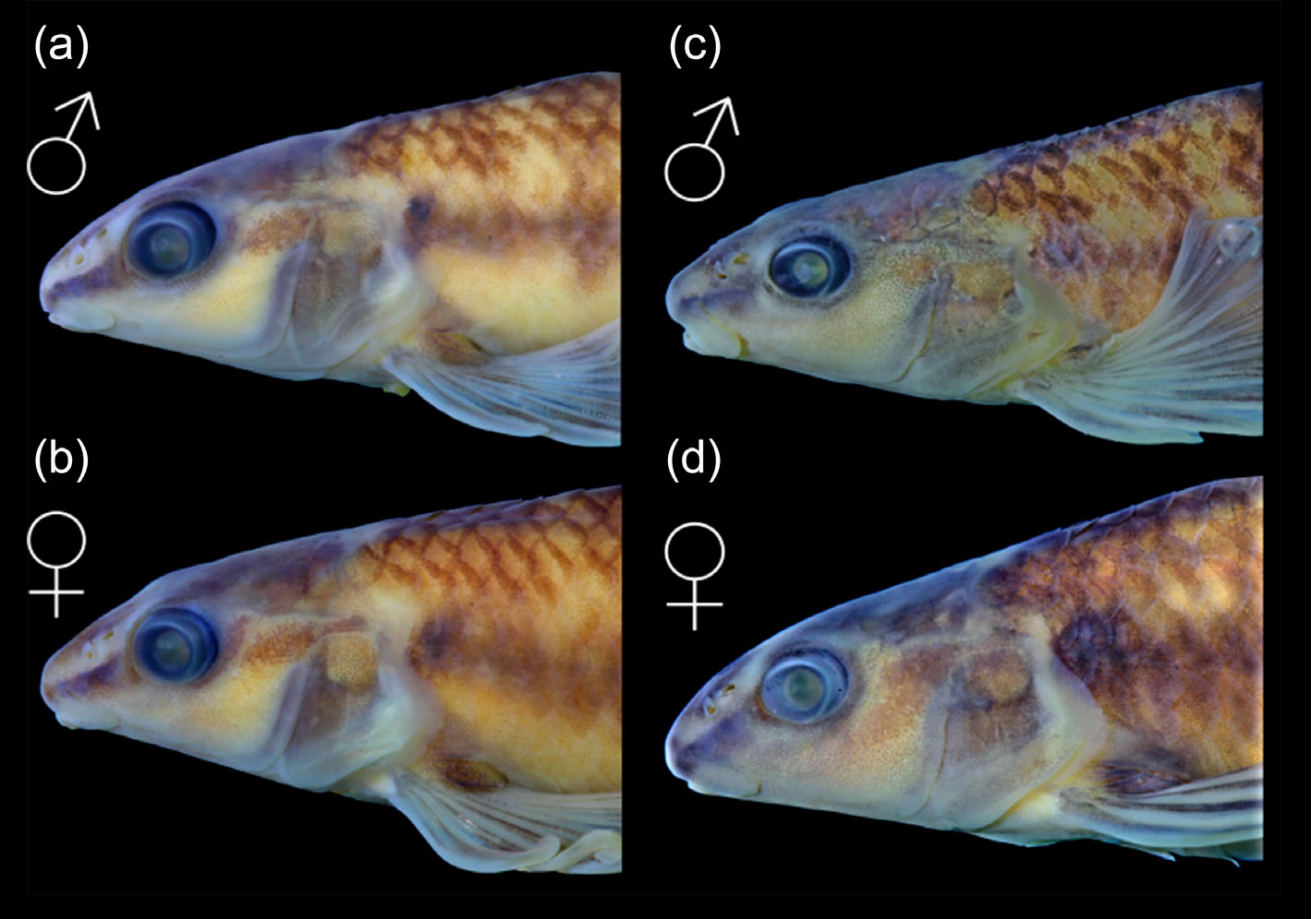
Lateral view of head of (a, b) *Characidium tupi* UFBA 8722, paratypes, rio Itanhém basin, Bahia state, male 50.7 mm *L*_S_ and female, 57.0 mm *L*_S_, and (c, d) *Characidium alipioi* UFBA 9939, rio Pomba, rio Paraíba do Sul basin, Minas Gerais state; male 52.5 mm *L*_S_ and female 56.0 mm *L*_S_.

**FIGURE 5:**
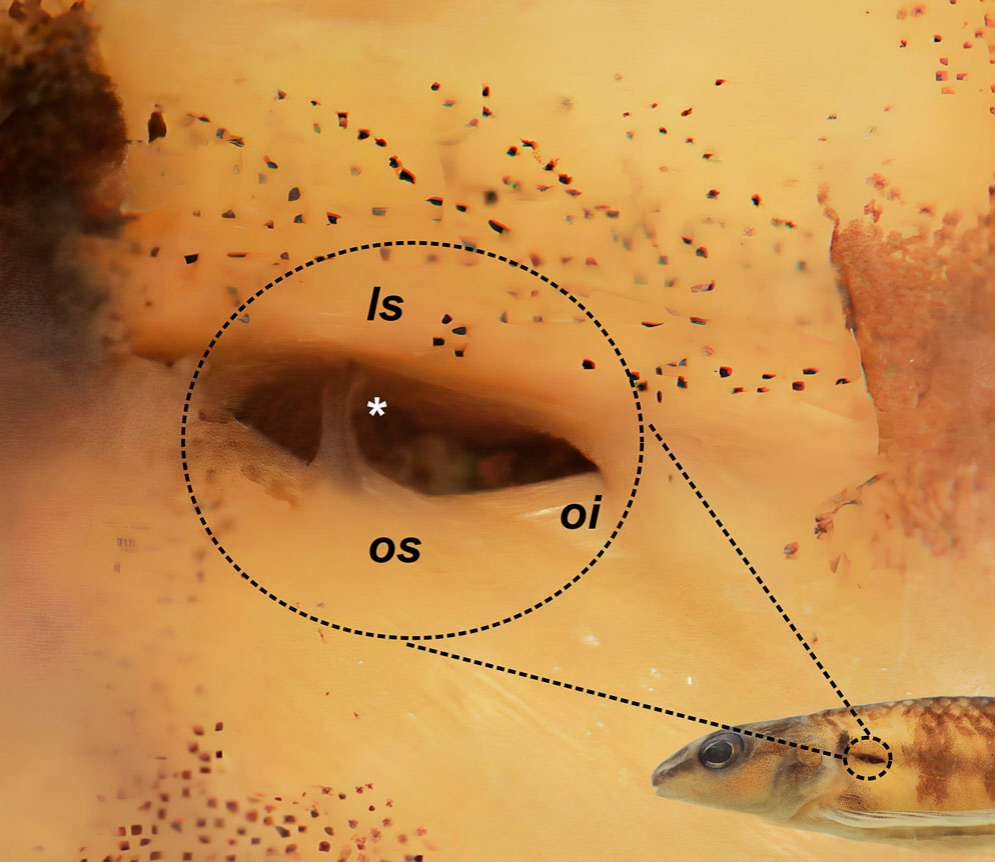
Pseudotympanum of *Characidium tupi* UFBA 8766, 58.9 mm *L*_S_, paratype, lateral view, right side. Overlying skin, adipose tissue and lateral‐line nerve removed. os, *obliquus superioris*; oi, *obliquus inferioris*; ls, *lateralis superficialis*. The asterisk indicates the rib of the fifth vertebra.

**FIGURE 6:**
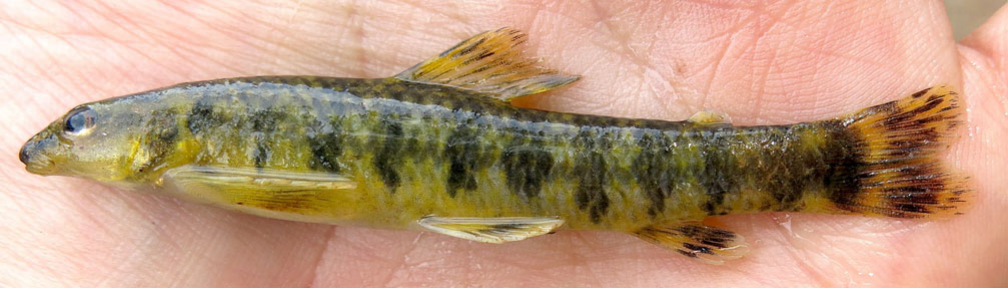
*Characidium tupi*, paratype, UFBA 8766, 62.8 mm *L*_S_, lateral view, freshly preserved specimens, rio dos Frades basin, Bahia state.

Figures 1, 2, 3, 4, 5, 6.

*Characidium* sp. 4.—Sarmento‐Soares & Martins‐Pinheiro, 2009: Figure 3 G [rio dos Frades, list of species].

*Characidium* sp. 1.—Oliveira‐Silva et al. (2024): Figure 2 p [populations reported from the Buranhém, Frades, Jucuruçu, and Itanhém rivers, molecuar species delimitation].

#### 3.1.1 Holotype

MZUSP 130914, 71.7 mm *L*_S_, Brazil, Bahia, Teixeira de Freitas, rio Itanhém, Povoado Prainha, near roadway BR 101, 17°30′09″S, 39°41′58.8″W, 26 m above sea level (m.a.s.l.), 14 January 2019, A. M. Zanata, R. Burger, G. V. Oliveira, L. Oliveira‐Silva.

Paratypes. All from Brazil. Bahia State. Rio Buranhém basin: UFBA 4706*, 15, 40.7–57.0 mm *L*_S_, Eunápolis, rio Buranhém close to the INCRA settlement behind Fazenda Japonesa, 16°22′39″S, 39°34′49″W, 24 October 2008, R. Burger, J. A. Reis. UFBA 4713*, 7, 29.1–56.1 mm *L*_S_, Eunápolis, rio Buranhém, Povoado Colônia, 24 October 2008, R. Burger, J. A. Reis. UFBA 4727*, 1, 42.4 mm *L*_S_, Eunápolis, tributary of the rio Buranhém near the Povoado Colônia, 24 October 2008, R. Burger, J. A. Reis. UFBA 4736, 1, 62.3 mm *L*_S_, Eunápolis, rio Buranhém, 24 October 2008, R. Burger, J. A. Reis. UFBA4939, 7, 45.5–53.0 mm *L*_S_, rio Buranhém near the Povoado Colônia, 16°15′41″S, 39°35′23″W, 140 m.a.s.l., 26 February 2009, A. M. Zanata, R. Burger, A. B. A. Góes, T. A. Carvalho. Rio dos Frades basin. UFBA 4955, 4, 40.6–54.6 mm *L*_S_, Itabela, Jacarandazinho stream, tributary of the rio dos Frades, on the border between Eunápolis and Itabela, 16°28′15″S, 39°34′32″W, 101 m.a.s.l., 26 February 2009, A. M. Zanata, R. Burger, A. B. A. Góes, T. A. Carvalho. UFBA 4962, 12, 28.1–46.4 mm *L*_S_, Itabela, rio dos Frades on roadway BR 101, 5 km south of Itabela, 16°37′00″S, 39°32′32″W, 66 m.a.s.l., 26 February 2009, A. M. Zanata, R. Burger, A. B. A. Góes, T. A. Carvalho. UFBA 8766*, 16, 27.2–62.4 mm *L*_S_, 1 c&s, 54.9 mm *L*_S_; MZUSP 130916, 8, 27.8–60.9 mm *L*_S_, Itabela, rio dos Frades, on roadway BR 101, 5 km south of Itabela, 16°37′01.0″S, 39°32′26″W, 52 m.a.s.l., 15 January 2019, A. M. Zanata, R. Burger, G. V. Oliveira, L. Oliveira‐Silva. UFBA 9947, 4, 31.3–40.2 mm *L*_S_, Rio Itanhém basin: UFBA 5064*, 4, 32.3–58.1 mm *L*_S_, Teixeira de Freitas, rio Itanhém, on Povoado Prainha, close to BR 101, 17°30′09″S, 39°41′59″W, 11 m.a.s.l., 28 February 2009, A. M. Zanata, R. Burger, A. B. A. Góes, T. A. Carvalho. UFBA 5077, 12, 34.5–49.8 mm *L*_S_, Teixeira de Freitas, rio Itanhetinga, on roadway BR 101, 17°21′10″S, 39°37′44″W, 46 m.a.s.l., 28 February 2009, A. M. Zanata, R. Burger, A. B. A. Góes, T. A. Carvalho. UFBA 8722*, 11, 23.6–61.6 mm *L*_S_, 1 c&s, mature female, 53.5 mm *L*_S_; MZUSP 130917, 5, 26.2–55.6 mm *L*_S_, collected with the holotype. Rio Jucuruçu basin. UFBA 4810, 10, 41.5–59.2 mm *L*_S_, Itamaraju, rio Jucuruçu, 25 October 2008, R. Burger, J. A. Reis. UFBA 4815*, 10, 42.0–67.2 mm *L*_S_; MZUSP 130918, 8, 39.0–71.0 mm *L*_S_, Itamaraju, rio Jucuruçu, 25 October 2008, R. Burger, J. A. Reis. UFBA 4819*, 22, 31.9–57.3 mm *L*_S_, 1 c&s, 57.0 mm *L*_*S*_; MZUSP 130919, 15, 38.6–46.9 mm *L*_S_, Vereda, stream of the rio Braço Sul, downstream of the Santa Clara dam, 26 October 2008, R. Burger, J. A. Reis. UFBA4826, 8, 39.5–48.9 mm *L*_S_, Vereda, rio Braço Sul, between Vereda and Itamaraju, downstream of the Santa Clara dam, 26 October 2008, R. Burger, J. A. Reis. UFBA 4831, 21, 33.0–42.3 mm *L*_S_, rio Braço Sul, between Vereda and Itamaraju, upstream of the Santa Clara dam, 26 October 2008, R. Burger, J. A. Reis. UFBA 4969*, 21, 30.7–56.9 mm *L*_S_, 1 c&s, mature male, 48.1 mm *L*_S_, Itamaraju, rio do Ouro, on roadway BR 101, 16°58′16″S, 39°33′07″W, 36 m.a.s.l., 26 February 2009, A. M. Zanata, R. Burger, A. B. A. Góes, T. A. Carvalho. Minas Gerais State: UFBA 9946, 115, 38–49.5 mm *L*_S_; MZUSP130920, 3, 46.2–49.6 mm *L*_S_, Palmópolis, rio Prado, rio Jucuruçu basin, 16°44′56″S, 40°25′50.4″W, 19 August 2022, A. M. Zanata, L. Oliveira‐Silva, R. Burger, T. Quadros. Espírito Santo State. **Rio São Mateus basin**: CZNC 307*, 10, 43.8–68.1 mm *L*_S_, Nestor Gomes, Cachoeira do Inferno, rio Cricaré, above and below the old dam, 18°42′27″S, 40°16′1″W, 30 October, 2012, L. F Duboc, L. F. S Ingenito, N. O. Sartor, P. A. S. Plesley, M. C. M. Sily, & I. M. Mazzini.

#### 3.1.2 Non types

UFBA 8744, 3, 52.7–67.7 mm *L*_S_, with spinal malformation, Brazil, Bahia, Eunápolis, rio Buranhém on rodway BR 101, rio Buranhém basin, 16°24′51.1″S, 39°35′10.1″W, 73 m.a.s.l., 13 January 2019, A. M. Zanata, L. Oliveira‐Silva, R. Burger, G. V. Oliveira. UFBA 10824, 5, 43.0–50 mm *L*_S_, alcohol fixation, mol, Brazil, Espírito Santo, Boa Esperança, rio Cotaxés, rio São Mateus basin, 18°34′24.4″S, 40°16′40.5″W, 16 m.a.s.l. 11 August 2022, A. M. Zanata, L. Oliveira‐Silva, R. Burger, T. Quadros.

#### 3.1.3 Diagnosis

*Characidium tupi* can be diagnosed from all congeners by its colour pattern, including 7–14 dark bars on laterals of the body, enlarged and more conspicuous along the midlateral line (Figures 1 and 2), resulting in a horizontal series of rounded or somewhat rectangle‐shaped blotches (vs. absence of dark bars or, if present, tapering ventrally and not forming conspicuous round or rectangle‐shaped dark blotches on the midlateral line). The new species can be further diagnosed from congeners, except *C. alipioi* Travassos, *C. amaila* Lujan, Agudelo‐Zamora, Taphorn, Booth & López‐Fernández, *C. boavistae* Steindachner, *C. bolivianum* Pearson, *C. crandellii* Steindachner, *C. cricarense, C. declivirostre* Steindachner, *C. duplicatum* Armbruster, Lujan & Bloom, *C. fasciatum* Reinhardt, *C. gomesi*, *C. grajahuense* Travassos, *C. helmeri*, *C. iaquira* Zanata, Ohara, Oyakawa & Dagosta, *C. japuhybense* Travassos, *C. kalunga* Melo, Bouquerel, Masumoto, França & Netto‐Ferreira, *C. kamakan*, *C. krenak*, *C. lauroi* Travassos, *C. nambiquara* Zanata & Ohara, *C. oiticicai* Travassos, *C. pterostictum* Gomes, *C. purpuratum* Steindachner, *C. schubarti* Travassos, *C. summus* Zanata & Ohara, *C. tatama* Agudelo‐Zamora, Tavera, Murillo & Ortega‐Lara, *C. timbuiense* Travassos, *C. travassosi* Melo, Buckup & Oyakawa, *C. vidali* Travassos and *C. wangyapoik* Armbruster, Lujan & Bloom by having the isthmus scaleless at least on its anteriormost portion (Figures 1 and 2) (vs. isthmus completely scaled). *Characidium tupi* further differs from *C. crandellii, C. declivirostre, C. duplicatum, C. iaquira* and *C. wangyapoik* by having the branchiostegal membranes free from each other and from the isthmus (Figure 3) (vs. membranes united to each other across isthmus), from *C. amaila*, *C. nambiquara* and *C. travassosi* by having 14 circumpeduncular scales (vs. 10–12 circumpeduncular scales), from *C. amaila*, *C. boavistae*, *C. bolivianum*, *C. fasciatum*, *C. gomesi*, *C. macrolepidotum*, *C. nambiquara* and *C. tatama* by having the isthmus and large areas around the bases of the pectoral‐fins scaleless but with central area between contralateral pectoral‐fin bases covered by scales (Figure 3) (vs. unscaled area extending from the isthmus to the whole area between the contralateral pectoral‐fin bases), from *C. helmeri* and *C. summus* by the adipose fin present (vs. adipose fin absent), from *C. cricarense* by having 3–4 scales between the anus and the anal‐fin origin (vs. 5–7 scales), from *C. kalunga* by having two series of dentary teeth (vs. one series), from *C. kamakan* by the absence of distinct black borders on scales forming short vertical black curved dashes on the body (vs. presence of short vertical black dashes on the borders of scales) and from *C. tatama* by having a 3‐shaped black band on the caudal fin (vs. caudal fin without black bands). *Characidium tupi* further differs from *C. gomesi*, *C. grajahuense*, *C. japuhybense*, *C. lauroi*, *C. oiticicai*, *C. pterostictum*, *C. purpuratum*, *C. schubarti*, *C. timbuiense* and *C. vidali* by having a dark band on the dorsal fin positioned below midlength of rays and initiating around the third or fourth branched rays (Figures 1 and 2) (vs. dark midlength band on dorsal fin, when present, initiating anteriorly, at the rear of the second unbranched or first branched ray). The new species further differs from *C. alipioi* by its overall more delicate head profile, with acuminate snout (Figure 4a,b) (vs. overall shape of the head robust, with a more stout snout Figure 4c,d), by a narrower cheek depth (12.0–13.9% of *L*_H_, mean = 12.9 and 1/3 to ¼ of the orbital diameter) (vs. 14.3–16.9%, mean = 15.4% of *L*_H_ and cheek less than half of the orbital diameter), by having patch of scales between contralateral pectoral‐fin bases extended anteriorly and expanded laterally, in front of the contralateral first pectoral‐fin rays (Figure 3a), or with at least some scales somewhat embedded in skin in that area (Figure 3b) (vs. scaled area between contralateral pectoral‐fin bases restricted to a triangular centralmost patch) (Figure 3c), rarely with two anteriorly positioned isolate scales (Figure 3d), and absence of the dimorphic colour pattern described by Serrano et al. (2018:75 and Figure 4) to *C. alipioi*. Besides morphological and morphometric characters, *C. tupi* can also be distinguished from the congeners by molecular divergence (see Results and Discussion for more details regarding morphological and molecular differences).

#### 3.1.4 Description

Morphometric data of holotype and paratypes in Table 1. Body elongate, somewhat cylindrical in cross‐section. Highest body depth at vertical through dorsal‐fin origin. Snout somewhat acuminate; dorsal profile of snout slight convex or straight in lateral view. Dorsal head profile nearly straight or slightly concave from snout to posterior naris, convex from that point to area above eyes and slightly convex or straight from that point to tip of supraoccipital. Dorsal body profile straight to slightly convex from tip of supraoccipital to end of dorsal‐fin base, straight from that point to adipose fin and slightly concave or straight from adipose fin to origin of anteriormost dorsal procurrent caudal‐fin rays. Ventral profile of head and body straight from dentary symphysis to isthmus, slightly convex from area between contralateral pectoral‐fin bases, slightly convex from that point to pelvic‐fin origin, straight or slightly concave from latter point to anal‐fin origin, concave along anal‐fin base and nearly straight or slightly concave from end of anal‐fin base to anteriormost ventral procurrent caudal‐fin ray. Head triangular in dorsal and lateral views; snout distinctly pointed. Mouth subterminal; anterior borders of premaxillary teeth visible in ventral view. Distal tip of maxilla distinctly short of the vertical through anterior margin of orbit. Orbit rounded, distinctly smaller than snout. Cheek somewhat broad, its depth a third to a fourth of orbit diameter. Nares separated, with contralateral margins raised; posterior naris closer to eye than to anterior naris. Supraorbital narrow, border abutting frontal convex and outer border slightly concave; anteriormost portion distant from frontal, resulting in angle with frontal. Nasal bones restricted to ossified canal. Parietal fontanel small, limited anteriorly by the parietals or small portion of the frontals and posteriorly by supraoccipital bones. Parietal branch of supraorbital canal present, exceeding frontal–parietal border and reaching at least midlength of the frontal.

**TABLE 1:** Morphometric data of holotype and paratypes of *Characidium tupi* (*n* = 35), range includes the holotype.

	Holotype	Range	Mean	SD
Total length (mm)	83.4	51.0–83.4	–	–
Standard length (mm)	71.7	41.3–71.7	–	–
Percents of standard length				
Depth at dorsal‐fin origin	18.4	18.4–23.6	20.9	1.6
Depth at anal‐fin origin	15.6	14.0–16.8	15.6	0.7
Caudal‐peduncle depth	10.7	10.1–11.7	11.0	0.4
Caudal‐peduncle length	16.5	15.2–18.6	17.1	0.9
Snout to dorsal‐fin origin	43.0	42.6–45.8	44.6	0.9
Snout to pectoral‐fin origin	21.5	20.0–23.3	21.7	0.8
Snout to pelvic‐fin origin	51.0	49.4–52.7	50.8	0.9
Snout to anal‐fin origin	77.7	74.8–78.1	76.4	0.8
Anal‐apex distance	92.6	90.7–98.2	93.5	1.4
Dorsal‐fin adpressed	26.1	25.4–29.0	27.3	0.8
Distance end dorsal to adipose	13.0	10.4–14.1	11.8	0.9
Pectoral‐fin length	23.0	23.0–28.6	25.5	1.6
Pelvic‐fin length	19.0	19.0–22.8	20.9	1.2
Anus to anal‐fin origin	7.9	6.8–10.2	8.6	1.0
Body width	12.1	12.1–18.5	14.4	1.6
Head length	22.9	20.0–24.2	22.9	1.0
Percents of head length				
Horizontal eye diameter	22.0	22.0–24.8	23.5	0.9
Snout length	29.9	27.8–30.8	29.3	0.8
Snout to maxillary tip	24.4	22.2–25.3	23.7	0.8
Anterior naris to orbit	9.8	9.7–12.4	11.1	0.7
Posterior naris to orbit	6.1	4.4–6.8	5.6	0.6
Cheek depth	13.4	12.0–13.9	12.9	0.6
Least interorbital width	18.9	18.6–21.0	19.5	0.7

Abbreviation: SD, standard deviation.

Dentary teeth in two rows; outer row with 8(8), 9(17), 10*(6) or 12 (1) teeth, anteriormost teeth tricuspid and posteriormost ones somewhat triangular and unicuspid; teeth decreasing in size from symphysis; inner row with several minute conical teeth inserted on edge of replacement tooth trench. Premaxilla with single row of 5*(13), 6(20) or 7(2) tricuspid teeth; teeth decreasing in size from symphysis. Maxillary edentulous. Ectopterygoid 6 (1), 8(1), 10(1) or 11(1) teeth arranged in one (4) row. Endopterygoid teeth absent. Branchiostegal rays 5(4), 4 connected to anterior ceratohyal, 1 connected to posterior ceratohyal.

Scales cycloid; *circuli* restricted to covered portion of scale; 15–25 parallel *radii* on exposed portion of scales of middle‐sized or large specimens (*c*. 60.0 mm *L*_S_). Lateral line slightly decurved, completely pored, with 35(11) or 36*(24) scales; horizontal scale rows above lateral line 4(10) or 5*(25); horizontal scale rows from lateral line to midventral scale series 3(5) or 4*(30). Scales along middorsal line between supraoccipital and origin of dorsal fin 9*(15) or 10(20). Scale rows around caudal peduncle 13(1) or 14*(34). Three (13), 4*(20) or 5(2) scales between anus aperture and anal‐fin insertion. Ventral surface of body without scales on anteriormost portion of isthmus and large areas around pectoral‐fin bases; patch of scales between contralateral pectoral‐fin bases extended anteriorly and expanded laterally, in front of the contralateral first pectoral‐fin rays, or with at least some scales somewhat embedded in skin in that area (Figure 3). Pseudotympanum as a *muscular* hiatus at vertical through anterior portion of swimbladder, represented by distinctly narrow opening partially anterior and partially posterior to rib of fifth vertebrae (Figure 5). Swimbladder with both chambers distinctly reduced; anterior chamber somewhat rounded, distinctly narrow in lateral view, depressed, completely attached to the Weberian Apparatus; posterior chamber elongate in males but vestigial in females. In males total length of swimbladder 10.3%–13.7% of *L*_S_ (3 specimens, 39.7–43.9 mm *L*_S_); length of anterior chamber 28.3%–43.5% and posterior chamber 56.5%–71.7% of swimbladder length. In females, total length of swimbladder 5.6%–9.3% of *L*_S_ (4 specimens, 42.8–56.3 mm *L*_S_); length of anterior chamber 42.5%–60.0% and posterior chamber 40.0%–57.5% of swimbladder length.

Dorsal‐fin rays ii8(1) or ii9*(34); distal margin of dorsal fin straight to slightly convex. Adipose fin well developed. Pectoral‐fin rays iii9i*(12), iii9ii(2), iii10(3), iii10i(16) or iii11(2); second to fourth branched pectoral‐fin rays usually longest; distal margin of pectoral rounded; posterior tip of pectoral fin not reaching pelvic‐fin insertion. Pelvic‐fin rays ii6i(2), i,7,i(25) or i8*(8); second or third branched pelvic‐fin rays longest; distal margin of fin rounded; posterior tip of pelvic fin not reaching anal‐fin origin. Anal‐fin rays ii,6*(34) or iii,5(1); first and second branched rays usually longest; distal margin of fin nearly straight to slightly concave; first anal‐fin radial between fifth caudal vertebrae (2); fin elements on last pterygiophore 2(30). Caudal‐fin rays i,8,i (1), i,9,7i *(3) or i,9,8,i(30); lobes similar in size. Dorsal procurrent caudal‐fin rays 8(3) or 9(1); ventral procurrent caudal‐fin rays 6(3) or 7(1). Total vertebrae 33(1), 34 (1) or 35(2); precaudal vertebrae 16 (1), 17(2) or 18(1); caudal vertebrae 17(3) or 18(1). Supraneural bones 3(1) or 5(3). Epural bones 2(4). Uroneural bone 1(4).

#### 3.1.5 Colour in alcohol

Ground colour of head and body pale yellow or light brown (Figures 1, 2 and 4). Head dorsum dark, usually with clear stretches from orbit to tip of snout, along nares, and clear area around posterior tip of the supraoccipital bone; some specimens darker than others and without clear stretches. Head with dark stripe from anterior margin of snout to anterior margin of eye, in lateral view; dark melanophores usually form an irregular blotch behind eyes somewhat continuous with stripe of snout. Opercle usually mostly dark. Ventral half of head, in lateral view, distinctly clearer than dorsal half, with sparse small melanophores. Ventral surface of head slightly darkened by scattered distributed small melanophores. Humeral blotch small, round, usually somewhat darker than midlateral blotches, with inconspicuous border and positioned immediately on the rear of opercle. Laterals of body with 7–14 dark bars enlarged and more conspicuous ventrally, resulting in a longitudinal series of conspicuous rounded or somewhat rectangle‐shaped blotches, extending from rear of opercle to end of caudal peduncle; wider portion of blotches usually over series of laterosensorial line of scales or over series immediately above it. Distance between blotches varies between specimens according to number of bars present, being narrower in specimens with higher number of bars. Variation in number of bars apparently independent from ontogeny, although highest counting more common on small specimens. Dorsalmost portion of bars more evident in specimens up to 50.0 mm *L*_S_, usually fading throughout ontogeny. Bars reaching or not ventral half of body, with variation throughout ontogeny; usually reaching in smaller specimens, more visible posterior to the pelvic fins (Figure 2c) and absent or reaching shortly below series of lateral line scales in larger specimens. Dorsal half of body usually distinctly darker than portion below lateral line; scales of three or four dorsalmost portion of body usually with melanophores concentrated on its distal half, forming oblique or longitudinal series of small dark blotches, resulting in inconspicuous dark longitudinal stripes. Inconspicuous saddle‐like dark blotches visible in dorsal view, usually more evident on posterior half of body, connected or not with lateral bars. Basicaudal black spot not visible. Ventral surface of body on isthmus and between pectoral‐fin contralateral bases clearer than surroundings, with a few sparse tiny melanophores; area posterior to pelvic fins darker than anterior portion in some specimens. All fins with dark pattern of pigmentation. Dorsal fin with base of anteriormost rays and membranes dark and two dark bands crossing fin: one dark band positioned below midlength of fin‐rays and initiating around the third or fourth branched rays and interradial membranes and reaching last fin ray; second band more distally positioned, initiating on second unbranched and reaching last branched ray; distal band usually wider than proximal band and distinctly darker on rays than on membranes. Caudal fin with a conspicuous 3‐shaped black band positioned approximately at midlength of lobes or slightly posterior; central portion of band extending anteriorly and covering three or four median caudal‐fin rays towards its base; blotch darker on rays but usually with melanophores also on membranes. From anteroposterior view each caudal‐fin lobe is dark at base of rays, followed by a clear area, a dark portion due to its half portion of the 3‐shaped blotch and a distally clear area, which may or not have dark traces. Anal fin with dark band crossing posterior half of rays and interradial membranes; basal portion of rays and interradial membranes dark in some specimens. Pectoral and pelvic fins similarly pigmented, with concentration of melanophores on dorsal portion of rays, forming or not a wide band crossing distal half of rays; melanophores rarely on interradial membranes. Some specimens with the whole body darker and bands on fin‐rays somewhat broader (Figure 2a). Adipose fin without conspicuously marked black pigment, usually with its posterodorsal border slightly darkened.

#### 3.1.6 Colour in life

Dark pattern on scales and fins similar to that of specimens in alcohol, although with dark midlateral blotch less evident. Ground colour of body yellowish, except by whitish ventral surface. Background of fins distinctively yellow. Dark marks on dorsal, anal, adipose and caudal fins conspicuous (Figure 6).

#### 3.1.7 Sexual dimorphism

No bony processes (spines or ridges; sensu Teixeira & Melo, 2021), or other external dimorphic feature were observed on fins or body of the specimens examined. Internally, both sexes possess swimbladder chambers distinctly reduced, vestigial, apparently more reduced in females than in males (total length 5.6%–9.3% of *L*_S_ vs. 10.3%–13.7%, respectively; see Description). The first chamber is similarly reduced in both sexes and the main observed difference between sexes is due to the length of the second chamber, shorter in females than in males (40.0%–57.5% vs. 56.5%–71.7% of swimbladder length). A confirmation of the swimbladder length variation as sexually dimorphic awaits a study focused on the matter and examination of a large number of specimens to the feature.

#### 3.1.8 Distribution

**FIGURE 7:**
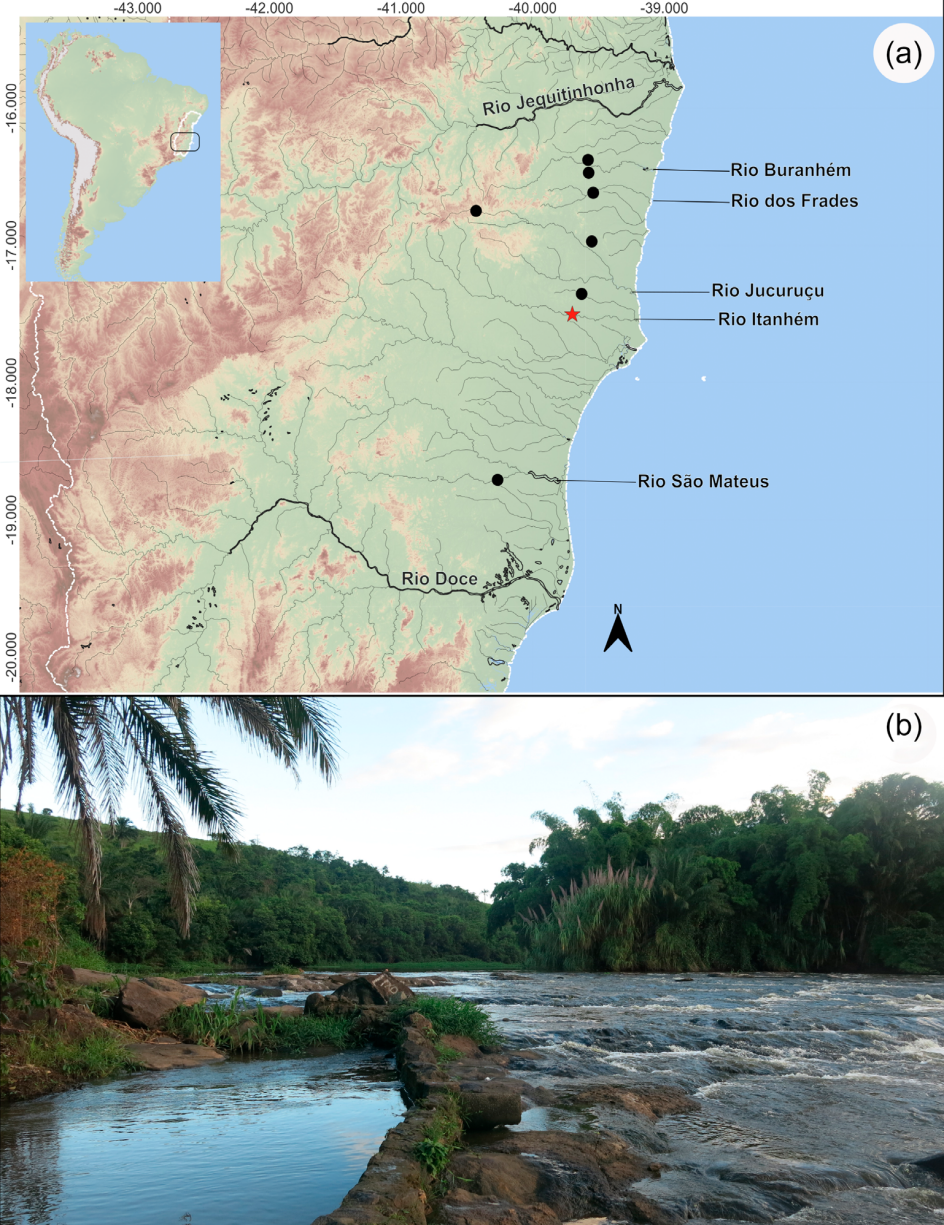
(a) Map with distribution of *Characidium tupi*, showing the type locality (red star) of and localities sampled; each symbol may represent more than one locality. (b) Type locality of *Characidium tupi*, Bahia, Teixeira de Freitas, rio Itanhém, near Povoado Prainha.

*Characidium tupi* is known from a series of relatively small independent eastern Brazilian drainages, between two relatively large river basins, the rio Jequitinhonha to the north and rio Doce to the south. Thus, the species is known from rio Buranhém, rio dos Frades, rio Itanhém, rio Jucuruçu and rio São Mateus (Figure 7a), with headwaters in the Minas Gerais State (except rio dos Frades) and meeting the Atlantic Ocean in the south of the Bahia State or north of the Espírito Santo State (i.e. rio São Mateus).

#### 3.1.9 Habitat and ecological notes

The area of occurrence of *Characidium tupi* is within the NMAF ecoregion (sensu Abell et al. (2008)). The original Atlantic Forest surrounding the rivers inhabited by the species had been converted into cocoa plantations in some stretches, and cattle ranches, coffee and rubber crops in others. *Characidium tupi* was sampled in streams with transparent or slightly dark waters, a few metres wide, in environments with fast water current over substrate composed of medium to large sized stones or rocks (Figure 7b). The species was found to occur syntopically with the congener *Characidium cricarense*, sampled together in the rivers Itanhém, Jucuruçu, and São Mateus.

#### 3.1.10 Etymology

The specific name *tupi* honours Tupi, as a legendary character from the Tupi‐Guarani mythology, the Tupi as a root to many indigenous nations within Brazil (e.g. Tupiniquim, Tupinambá), and also honours the Tupi as an old language, heard by the sailors when Pedro Álvares Cabral's fleet arrived in Brazil around 1500 and currently considered one of the most important in the spiritual and cultural construction of the country (Fausto, 2010; Navarro, 2013). A noun in apposition.

### 3.2 Molecular analyses

**FIGURE 8:**
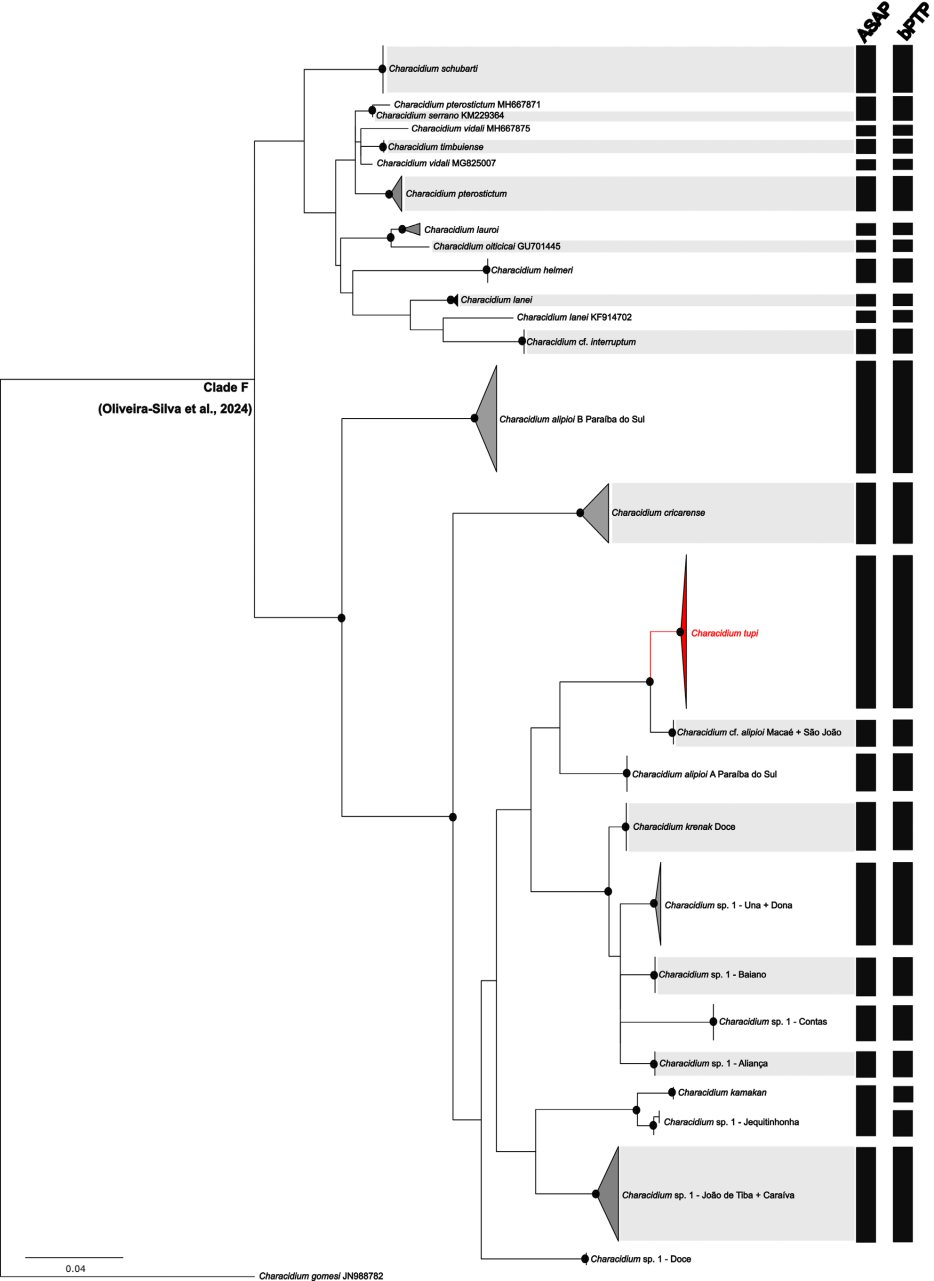
Maximum likelihood phylogenetic tree of *Characidium* species inferred from cytochrome c oxidase subunit I (COI) gene sequences. Black circles indicate bootstrap support values greater than 80. Black vertical bars represent species delimitated by the assemble species by automatic partitioning (ASAP) and Poisson tree process (bPTP) analyses.

The molecular dataset consisted of 105 sequences, each 637 base pairs long, with 141 variable sites. The Iss values were lower than the Iss.c values, indicating no evidence of saturation in the dataset. The ML tree showed high bootstrap values supporting each of the analysed species (Figure 8). The best partition proposed by ASAP identified 25 molecular operational taxonomic units (ASAP score 2.00), supporting *C. tupi* as a new species. Notably, the bPTP analysis using ML produced nearly identical results, except for sequences of *C. kamakan* and *Characidium* sp. 1 from the rio Jequitinhonha, which were classified as distinct species (Figure 8), whereas ASAP grouped them as a single species. The interspecific genetic distance within the *Characidium* ‘clade F’ ranged from 2%, as observed between *Characidium* sp. 1 from the rio Baiano and *Characidium* sp. 1 from the Una and Dona rivers, to 18%, as seen between *C. krenak* and *C*. cf. *interruptum* (Table S2).

## 4 DISCUSSION

*Characidium tupi* shares the three potential synapomorphies proposed by Buckup (1993b) to Clade C1: the poscleithrum 1 reduced or absent (absent in the species), a scaleless area on the isthmus and the reduction of the fontanel and exclusion of the frontals of its anterior margin. Seven species were originally included in Clade C1 (i.e. *C. boavistae*, *C. bolivianum*, *C. crandellii*, *C. declivirostre*, *C. fasciatum*, *C. gomesi*, *C. purpuratum*). Posteriorly, a series of other species were tentatively hypothesized as members of that clade (i.e. *C. alipioi*, *C. amaila*, *C. cricarense*, *C. dule*, *C. grajahuense*, *C. helmeri*, *C. japuhybense*, *C. kamakan*, *C. krenak*, *C. lauroi*, *C. macrolepidotus*, *C. oiticicai*, *C. pterostictum*, *C. purpuratum*, *C. schubarti*, *C. tatama*, *C. travassosi*, *C. timbuiense* and *C. vidali*), based on the shared morphological features or results of molecular phylogeny (Buckup & Reis, 1997; Lujan et al., 2013; Zanata & Camelier, 2015; Melo et al., 2016; Agudelo‐Zamora et al., 2020; Oliveira‐Silva et al., 2022). However, broad variation on the pattern of scalation of the isthmus occurs within the group of species cited, along with variation in overall body shape and other morphological characters, rendering questionable the close relationships of all species putatively included in the Clade C1. *Characidium tupi* also shares overall shape of body and fins related to congeners that inhabit fast‐flowing water environments with the species from the clade C1, and some other congeners, as discussed by various authors (e.g. Buckup et al., 2000; Oliveira‐Silva et al., 2022; Zanata & Camelier, 2015; Zanata & Ohara, 2020).

Species delimitation analyses further reinforced the recognition of *C. tupi* as a distinct taxon. The results identified 25 or 26 species within clade F (as per ASAP and PTP methods, respectively), with both methods consistently supporting *C. tupi* as a new species (Figure 8). The slight discrepancy between ASAP and PTP—specifically in the delimitation between *C. kamakan* and *Characidium* sp.1 from the rio Jequitinhonha—may stem from differences in algorithmic approaches, population sizes, species diversity and speciation rates (Puillandre et al., 2021). These factors influence species delimitation within this clade and highlight the need for further investigation, especially considering the apparent presence of cryptic diversity. Overall, species delimitation methods are valuable tools that, when integrated with additional lines of evidence, such as morphological data, strengthen the support for species boundaries (Stabile et al., 2025; Zanata et al., 2024). Moreover, the taxonomic limits of *C. tupi* were defined with a high degree of confidence, as our dataset includes a comprehensive sampling of sequences and populations covering the entire distribution of the new species, as well as other closely related species—an essential consideration when working with complex taxonomic groups (Morando et al., 2003). This robust approach confirms that these populations share a common evolutionary history, as evidenced by recovered phylogenetic relationships.

The phylogenetic results are consistent with those presented by Oliveira‐Silva et al. (2024), in which populations previously assigned to *Characidium* sp.1 (from the Buranhém, Frades, Jucuruçu, and Itanhém rivers)—now described here as a new species, including populations from the rio São Mateus—are closely related to populations assigned to *C. alipioi* within clade F. Serrano et al. (2018) questioned the taxonomic delimitation of *C. alipioi* due to the genetic structuring observed among its populations. According to the authors, species delimitation using mitochondrial data strongly supports the presence of two sympatric species within *C. alipioi* in the rio Paraíba do Sul (i.e. their *C. alipioi* A and *C. alipioi* B), despite limited morphological variation and conserved chromosomal patterns. The genetic distances obtained in the present study support the recognition of *C. tupi* as a distinct species, with divergences exceeding 6% in relation to *C*. aff. *alipioi* A and 13% in relation to *C*. aff. *alipioi* B from the rio Paraíba do Sul (Table S2). Nonetheless, while the delimitation of *C. tupi* is well‐supported, additional approaches, such as multilocus or genomic studies focused on *C. alipioi* (ongoing projects led by LOS), are necessary to further explore the diversification history of these species, along the Brazilian coast. Such studies could uncover even greater hidden diversity, including the potential existence of yet undescribed species, which likely diverged very recently—a pattern already reported for several fish groups along the Brazilian coastal region (Oliveira‐Silva et al., 2023; Poveda‐Martínez et al., 2016; Santos et al., 2021). Morphological features promptly differentiate *Characidium tupi* from C. *alipioi* (A or B; see Diagnosis), including distinct coloration pattern, including conspicuous dark midlateral blotches in the former, distinct head structure (e.g. slender head profile, cheek narrower), distinct pattern of scales covering area between isthmus and pectoral‐fin bases and absence of the dimorphic colour pattern described by Serrano et al. (2018:75 and Figure 4) to *C. alipioi*.

*Characidium tupi* possesses a vestigial swimbladder, with both chambers distinctly reduced, although slightly longer in the males examined (see Description section). In fact, the second chamber of the swimbladder is apparently sexually dimorphic, longer in males than in females. In their review of the secondary sexual characters Teixeira and Melo (2021) reported three types of sexual dimorphism occurring in species of *Characidium,* which include bony processes on fin rays, size and shape of the anal and pelvic fins, and sexual dichromatism of body and fins. Among the congeners examined, specimens of *C. cricarense* revealed a somewhat similar condition to that observed in *C. tupi*, having the second chamber of males longer than that of the females. Most of the congeners examined for this feature do not present dimorphic condition of the swimbladder, but a few specimens were examined for this feature and a comprehensive analysis (ongoing projects led by A.M.Z.) is necessary to confidently define the variation as sexually dimorphic.

## 5 COMPARATIVE MATERIAL EXAMINED

Comparative material was obtained from the list of species provided by Zanata et al. (2018, 2023), with addition of *Characidium alipioi* LPB 8378, 25, 36.5–66.1 mm *L*_S_, rio Paraíba do Sul basin; LBP 25739, 3, 52.1–70.2 mm *L*_S_, rio Paraíba do Sul basin; LBP 25740, 5, 71.4–92.1 mm *L*_S_, rio Paraíba do Sul basin; MZUSP 126715, 5, 31.3–68.0 *L*_S_, rio Bananal, rio Paraíba do Sul basin; MZUSP 126723, 5, 46.8–74.0 mm *L*_S_, tributary of rio Preto, rio Paraíba do Sul basin; UFBA 9936, 11, 24.378.7 mm *L*_S_, tributary of rio Muriaé, rio Paraíba do Sul basin; UFBA 9939, 38, 45.6–69.5 mm *L*_S_, rio Pomba, rio Paraíba do Sul basin; MZUSP 124820, 10, 43.4–74.0 mm *L*_S_, rio Paraíba do Sul basin. *Characidium cricarense* CZNC 1587, 78, 25.3–46.6 mm *L*_S_, rio São Mateus basin; MNRJ 50973, paratypes, 16, 39.3–48.0 mm *L*_S_, rio São Mateus basin; UFBA 8742, 5, 24.8–31.1 mm *L*_S_, rio Jucuruçu basin; MNRJ 41832, paratypes, 23, 40.6–55.7 mm *L*_S_, rio Doce basin. *Characidium geryi* MUSM 21873, 10, 13.6–19.5 mm *L*_S_, 1 c&s, 17.9 mm *L*_S_, río Marañón basin, Peru; MUSM 41993, 1, 25.8 mm *L*_S_, río Marañón basin. *Characidium interruptum* UFBA 10006, 7, 30.4–36.6 mm *L*_S_, rio São João. *Characidium littorale* UFBA 10008, 24, 25.6–43.2 mm *L*_S_, rio São João. *Characidium* cf. *pellucidum* MZUSP 118834, 25, 17.4–23.0 mm *L*_S_, rio Madeira basin; MZUSP 121994, 7, 26.1–28.9 mm *L*_S_, rio Madeira basin. *Characidium sterbai* UFBA 10825, 6, 24.6–30.7 mm *L*_S_ S, río Ucayali basin. *Characidium timbuiense* UFBA 9938, 57, 20.0–49.6 mm *L*_S_, rio Timbuí, rio Reis Magos basin. *Characidium* cf. *vidali* UFBA 9942, 42, 40.6–71.9 mm *L*_S_, rio do Ouro, rio Macaé basin.

## AUTHOR CONTRIBUTIONS

A.M.Z. contributed substantially to the acquisition, analysis and interpretation of data, and the writing of the initial and subsequent drafts of the manuscript. L.O.S. contributed substantially to the study's conception and design, data acquisition and analysis, manuscript writing and critical review for important intellectual content.

## CONFLICT OF INTEREST STATEMENT

This study was done without conflict of interest.

## Supporting information


**TABLE S1.** Taxa, vouchers, locality and GenBank accession numbers of specimens of *Characidium* used in the mitochondrial DNA analysis. The acronyms of institutions follow Fricke et al. (2025).

**TABLE S2.** Genetic distances and standard deviation for the species analysed in the study.
