# Supplementary material for: A new South American darter (Crenuchidae: Characidium ) from rivers draining the Northeastern Mata Atlantica Freshwater Ecoregion, Brazil: morphological and molecular evidence
Source: J Fish Biol. 2025 Dec 23;108(4):1311–23. doi: 10.1111/jfb.70316 (PMC13193469; doi:10.1111/jfb.70316)
Supplement: Supplementary file 1 — TABLE S1. Taxa, vouchers, locality and GenBank accession numbers of specimens of Characidium used in the mitochondrial DNA analysis. The acronyms of institutions follow Fricke et al. (2025). [file JFB-108-1311-s001.docx]

Table S1. Taxa, vouchers, locality and GenBank/BOLD accession numbers of specimens of *Characidium* used in the mitochondrial DNA analysis. The acronyms of institutions follow Fricke *et al*. ([202](https://onlinelibrary.wiley.com/doi/10.1111/jfb.15402#jfb15402-bib-0026)5)

| Taxon | Museum ID | Specimen | Locality | City, State | GenBank/BOLD n. |
| --- | --- | --- | --- | --- | --- |
| *C. alipioi* | MNRJ39301 | 5390 | Macaé | Macaé, Rio de Janeiro, BR | MH667901 |
| *C. alipioi* | MNRJ39301 | 5393 | Macaé | Macaé, Rio de Janeiro, BR | MH667874 |
| *C. alipioi* | MNRJ42780 | 9341 | São João | Silva Jardim, Rio de Janeiro, BR | MH667868 |
| *C. alipioi*  A | LBP 25740 | 78709 | Rio Paraíba do Sul | Pindamonhangaba, São Paulo | MH716095 |
| *C. alipioi*  A | LBP 25740 | 78712 | Rio Paraíba do Sul | Pindamonhangaba, São Paulo | MH716097 |
| *C. alipioi*  A | LBP 25740 | 78713 | Rio Paraíba do Sul | Pindamonhangaba, São Paulo | MH716098 |
| *C. alipioi*  A | LBP 25740 | 78750 | Rio Paraíba do Sul | Pindamonhangaba, São Paulo | MH716119 |
| *C. alipioi*  B | LBP 25739 | 78711 | Rio Paraíba do Sul | Pindamonhangaba, São Paulo | MH716122 |
| *C. alipioi* B | LBP 25739 | 78714 | Rio Paraíba do Sul | Pindamonhangaba, São Paulo | MH716123 |
| *C. alipioi* B | LBP 25739 | 78717 | Rio Paraíba do Sul | Pindamonhangaba, São Paulo | MH716124 |
| *C. alipioi*  B | LBP 25739 | 78726 | Rio Paraíba do Sul | Pindamonhangaba, São Paulo | MH716127 |
| *C. alipioi* B | LBP 25739 | 78728 | Rio Paraíba do Sul | Pindamonhangaba, São Paulo | MH716128 |
| *C. alipioi*  B | LBP 8378 | 40203 | Paraiba do Sul | Santa Barbara Tugurio, Minas Gerais, BR | HM064986 |
| *C. alipioi* B | LBP 8378 | 40204 | Paraiba do Sul | Santa Barbara Tugurio, Minas Gerais, BR | HM064987 |
| *C. alipioi* B | LBP 8378 | 40205 | Paraiba do Sul | Santa Barbara Tugurio, Minas Gerais, BR | HM064988 |
| *C. alipioi*  B | LBP 8378 | 40201 | Paraiba do Sul | Santa Barbara Tugurio, Minas Gerais, BR | GU702090 |
| *C. alipioi*  B | LBP 8378 | 40202 | Paraiba do Sul | Santa Barbara Tugurio, Minas Gerais, BR | GU702087 |
| *C. cricarense* | CZNC 1506 | 8162 | São Mateus | Água Doce do Norte, Espírito Santo, BR | MH667902 |
| *C. cricarense* | MNRJ 50973 | 8269 | São Mateus | Água Doce do Norte, Espírito Santo, BR | MH667896 |
| *C. cricarense* | MNRJ 41832 | 5314 | Doce | Santa Teresa, Espírito Santo, BR | MH667899 |
| *C. cricarense* | MNRJ 41832 | 5315 | Doce | Santa Teresa, Espírito Santo, BR | MH667878 |
| *C. cricarense* | MNRJ 41832 | 5316 | Doce | Santa Teresa, Espírito Santo, BR | MH667864 |
| *C. cricarense* | LBP 12260 | 52191 | Doce | Desterro do Melo, Minas Gerais, BR | PP725100 |
| *C.* cf. *interruptum* | UFBA 8709 | 2485 | Rio Cahy | Prado, Bahia, BR | PP725108 |
| *C.*  cf. *interruptum* | UFBA 8709 | 2486 | Rio Cahy | Prado, Bahia, BR | PP725109 |
| *C.*  cf. *interruptum* | UFBA 8711 | 2490 | Rio Cahy | Prado, Bahia, BR | PP725110 |
| *C. gomesi* | LBP 1181 | 10043 | Upper Paraná | Botucatu, São Paulo, BR | JN988782 |
| *C. helmeri* | MZUSP112666 | 585 | Rio Cahy | Prado, Bahia, BR | PP725111 |
| *C. helmeri* | MZUSP112653 | 587 | Rio Cahy | Prado, Bahia, BR | PP725112 |
| *C. helmeri* | UFBA 8715 | 2504 | Rio Cahy | Prado, Bahia, BR | PP725113 |
| *C. kamakan* | UFBA8765 | 2708 | Pardo | Camacan, Bahia, BR | PP725114 |
| *C. kamakan* | UFBA8765 | 2709 | Pardo | Camacan, Bahia, BR | PP725115 |
| *C. kamakan* | UFBA8765 | 2710 | Pardo | Camacan, Bahia, BR | PP725116 |
| *C. krenak* | - | LGC 5752 | Doce | Conceição do Mato Dentro, Minas Gerais, BR | BOLD:ACS9348 |
| *C. krenak* | - | LGC 5719 | Doce | Alvorada de Minas, Minas Gerais, BR | BOLD:ACS9348 |
| *C. krenak* | MCNIP 4889 | RD161 | Doce | Alvorada de Minas, Minas Gerais, BR | BOLD:ACS9348 |
| *C. krenak* | LGC 5742 | 5742 | Doce | Conceição do Mato Dentro, Minas Gerais, BR | BOLD:ACS9348 |
| *C. lanei* | - | - | - | - | KF914702 |
| *C. lanei* | - | - | - | - | KF914697 |
| *C. lanei* | MNRJ 40905 | 4970 | Guaraguaçu | Paranaguá, Paraná, BR | MG825016 |
| *C. lauroi* | LBP 8741 | 46417 | Costeiro do Atlântico | Ubatuba, São Paulo, BR | KM229366 |
| *C. lauroi* | LBP 8741 | 35722 | Costeiro do Atlântico | Ubatuba, São Paulo, BR | KF914701 |
| *C. oiticicai* | LBP 8703 | 31218 | Upper Paraná | Salesópolis, São Paulo, BR | GU701445 |
| *C. pterostictum* | MNRJ 41094 | 7614 | Tramandaí | Barra do Ouro, Rio Grande do Sul, BR | MH667871 |
| *C. pterostictum* | LBP 8700 | 33509 | Jacareí | Morretes, Paraná, BR | KF914710 |
| *C. pterostictum* | LBP 8701 | 33516 | Jacareí | Paranaguá, Paraná, BR | KF914709 |
| *C. pterostictum* | LBP 7367 | 33636 | Ribeira de Iguape | Iporanga, São Paulo, BR | KF914700 |
| *C. pterostictum* | LBP 7367 | 33671 | Ribeira de Iguape | Iporanga, São Paulo, BR | KF914708 |
| *C. schubarti* | LBP 8702 | 31492 | Upper Paraná | Jaguariaíva, Paraná, BR | GU701440 |
| *C. schubarti* | LBP 8702 | 31498 | Upper Paraná | Jaguariaíva, Paraná, BR | GU701439 |
| *C. schubarti* | LBP 8702 | 33512 | Upper Paraná | Jaguariaíva, Paraná, BR | GU701437 |
| *C. serrano* | LBP 19568 | 62715 | Uruguai River | Pirapó, Rio Grande do Sul, BR | KM229364 |
| *C. timbuiense* | MNRJ 41900 | 9077 | Reis Magos | Santa Tereza, Espirito Santo, BR | MH667892 |
| *C. timbuiense* | LBP 19565 | 69812 | Reis Magos | Santa Tereza, Espirito Santo, BR | KM229365 |
| *C. tupi* | UFBA8744 | 2636 | Buranhém | Eunápolis, Bahia, BR | PP725156 |
| *C. tupi* | UFBA8744 | 2637 | Buranhém | Eunápolis, Bahia, BR | PP725157 |
| *C. tupi* | UFBA8744 | 2638 | Buranhém | Eunápolis, Bahia, BR | PP725158 |
| *C. tupi* | UFBA8766 | 2715 | Rio dos Frades | Itabela, Bahia, BR | PP725159 |
| *C. tupi* | UFBA8766 | 2716 | Rio dos Frades | Itabela, Bahia, BR | PP725160 |
| *C. tupi* | UFBA8766 | 2717 | Rio dos Frades | Itabela, Bahia, BR | PP725161 |
| *C. tupi* | UFBA8743 | 2634 | Jucuruçu | Itamarajú, Bahia, BR | PP725167 |
| *C. tupi* | UFBA8743 | 2635 | Jucuruçu | Itamarajú, Bahia, BR | PP725168 |
| *C. tupi* | UFBA 8722 | 2525 | Itanhém | Teixeira de Freitas, Bahia, BR | PP725169 |
| *C. tupi* | UFBA 8722 | 2526 | Itanhém | Teixeira de Freitas, Bahia, BR | PP725170 |
| *C. tupi* | UFBA 10824 | 2870 | São Mateus | Boa Esperança, Espírito Santo, BR | PX508940 |
| *C. tupi* | UFBA 10824 | 2871 | São Mateus | Boa Esperança, Espírito Santo, BR | PX508941 |
| *C. tupi* | UFBA 10824 | 2872 | São Mateus | Boa Esperança, Espírito Santo, BR | PX508943 |
| *C. tupi* | UFBA 10824 | 2873 | São Mateus | Boa Esperança, Espírito Santo, BR | PX508942 |
| *C. vidali* | MNRJ 40284 | 6206 | Macacu | Guapimirim, Rio de Janeiro, BR | MH667875 |
| *C. vidali* | MNRJ39297 | 3071 | São João | Casimiro de Abreu, Rio de Janeiro, BR | MG825007 |
| *Characidium* sp. 1 | UFBA 8710 | 2487 | Una | Valença, Bahia, BR | PP725134 |
| *Characidium* sp. 1 | UFBA 8710 | 2488 | Una | Valença, Bahia, BR | PP725135 |
| *Characidium* sp. 1 | UFBA 8710 | 2489 | Una | Valença, Bahia, BR | PP725136 |
| *Characidium* sp. 1 | UFBA 7461 | 2609 | Rio da Dona | Varzedo, Bahia, BR | PP725129 |
| *Characidium* sp. 1 | UFBA 7461 | 2610 | Rio da Dona | Varzedo, Bahia, BR | PP725130 |
| *Characidium* sp. 1 | UFBA 7461 | 2611 | Rio da Dona | Varzedo, Bahia, BR | PP725131 |
| *Characidium* sp. 1 | UFBA 7461 | 2612 | Rio da Dona | Varzedo, Bahia, BR | PP725132 |
| *Characidium* sp. 1 | UFBA 8325 | 2606 | Rio da Dona | Varzedo, Bahia, BR | PP725133 |
| *Characidium* sp. 1 | UFBA 8738 | 2624 | Baiano | Maraú, Bahia, BR | PP725137 |
| *Characidium* sp. 1 | UFBA 8738 | 2625 | Baiano | Maraú, Bahia, BR | PP725138 |
| *Characidium* sp. 1 | UFBA 8738 | 2626 | Baiano | Maraú, Bahia, BR | PP725139 |
| *Characidium* sp. 1 | UFBA 8738 | 2627 | Baiano | Maraú, Bahia, BR | PP725140 |
| *Characidium* sp. 1 | UFBA8759 | 2690 | Contas | Iguaí, Bahia, BR | PP725141 |
| *Characidium* sp. 1 | UFBA8759 | 2691 | Contas | Iguaí, Bahia, BR | PP725142 |
| *Characidium* sp. 1 | UFBA8759 | 2692 | Contas | Iguaí, Bahia, BR | PP725143 |
| *Characidium* sp. 1 | UFBA8759 | 2693 | Contas | Iguaí, Bahia, BR | PP725144 |
| *Characidium* sp. 1 | UFBA8759 | 2694 | Contas | Iguaí, Bahia, BR | PP725145 |
| *Characidium* sp. 1 | UFBA8757 | 2683 | Aliança | Arataca, Bahia, BR | PP725146 |
| *Characidium* sp. 1 | UFBA8757 | 2684 | Aliança | Arataca, Bahia, BR | PP725147 |
| *Characidium* sp. 1 | UFBA8757 | 2685 | Aliança | Arataca, Bahia, BR | PP725148 |
| *Characidium* sp. 1 | UFBA6970 | 2615 | Jequitinhonha | Salinas, Minas Gerais, BR | PP725149 |
| *Characidium* sp. 1 | LBP 8289 | 38354 | Jequitinhonha | Salinas, Minas Gerais, BR | PP725150 |
| *Characidium* sp. 1 | LBP 8289 | 38355 | Jequitinhonha | Salinas, Minas Gerais, BR | PP725151 |
| *Characidium* sp. 1 | UFBA8745 | 2639 | João de Tiba | Eunápolis, Bahia, BR | PP725152 |
| *Characidium* sp. 1 | UFBA8745 | 2640 | João de Tiba | Eunápolis, Bahia, BR | PP725153 |
| *Characidium* sp. 1 | UFBA8745 | 2641 | João de Tiba | Eunápolis, Bahia, BR | PP725154 |
| *Characidium* sp. 1 | UFBA8745 | 2642 | João de Tiba | Eunápolis, Bahia, BR | PP725155 |
| *Characidium* sp. 1 | UFBA 8714 | 2493 | Rio Caraíva | Itabela, Bahia, BR | PP725162 |
| *Characidium* sp. 1 | UFBA 8714 | 2494 | Rio Caraíva | Itabela, Bahia, BR | PP725163 |
| *Characidium* sp. 1 | UFBA 8714 | 2495 | Rio Caraíva | Itabela, Bahia, BR | PP725164 |
| *Characidium* sp. 1 | UFBA 8714 | 2496 | Rio Caraíva | Itabela, Bahia, BR | PP725165 |
| *Characidium* sp. 1 | UFBA 8714 | 2497 | Rio Caraíva | Itabela, Bahia, BR | PP725166 |
| *Characidium* sp. 1 | UFBA 9940 | 3300 | Doce | Senador Firmino, BR | PX508938 |
| *Characidium* sp. 1 | UFBA 9940 | 3301 | Doce | Senador Firmino, BR | PX508939 |
|  |  |  |  |  |  |
| *C. kamakan* | UFBA8765 | 2708 | Pardo | Camacan, Bahia, BR | PP725114 |
| *C. kamakan* | UFBA8765 | 2709 | Pardo | Camacan, Bahia, BR | PP725115 |
| *C. kamakan* | UFBA8765 | 2710 | Pardo | Camacan, Bahia, BR | PP725116 |
